# Assessing the Impact of Shredded Polyethylene Terephthalate (PET) Post-Consumer Plastic as a Partial Replacement for Coarse Aggregates in Unreinforced Concrete

**DOI:** 10.3390/ma17215208

**Published:** 2024-10-25

**Authors:** Elias Farah, Saidé Yaacoub, Joseph Dgheim, Nemr El Hajj

**Affiliations:** 1 Department of Civil Engineering, School of Engineering, Holy Spirit University of Kaslik (USEK), Jounieh P.O. Box 446, Lebanon; saide.y.yaacoub@net.usek.edu.lb; 2 Group Mechanical Thermal and Renewable Energies, GMTER, Faculty of Sciences II, Lebanese University, Fanar P.O. Box 90656, Lebanon; jdgheim@ul.edu.lb; 3 Department of Mechanical Engineering, School of Engineering, Holy Spirit University of Kaslik (USEK), Jounieh P.O. Box 446, Lebanon; nemrelhajj@usek.edu.lb

**Keywords:** coarse aggregate replacement, compressive strength, concrete, masonry blocks, PET, thermal

## Abstract

This study investigates the feasibility of incorporating shredded polyethylene terephthalate (PET) post-consumer plastic waste as a partial replacement for coarse aggregates in unreinforced concrete such as masonry blocks. Standard concrete blocks were produced with varying PET content (0%, 5%, 25%, 35%, 50%) and tested for workability, air content, density, compressive strength, flexural strength, and thermal conductivity. Results indicated that replacing up to 25% of traditional aggregates with PET maintains adequate compressive strength for non-load-bearing applications and enhances thermal insulation by reducing the thermal conductivity from 0.7 W/m·°K to 0.27 W/m·°K at 25% replacement level, representing a significant improvement of approximately 61%. Higher PET content (35–50%) resulted in reduced structural integrity but improved insulation, suggesting its suitability for non-structural applications. This research highlights the potential of using PET plastic waste in unreinforced concrete, promoting sustainable construction practices by reducing plastic waste and conserving natural resources.

## 1. Introduction

The construction industry continuously seeks sustainable practices to mitigate its environmental impact while maintaining the structural integrity and performance of building materials. Masonry blocks are traditionally composed of sand, cement, water, and fine aggregate. However, environmental concerns necessitate innovative approaches to minimize ecological impacts while maintaining material performance.

Plastic waste, particularly polyethylene terephthalate (PET), poses significant environmental challenges due to its durability and resistance to degradation. Globally, over 350 million tons of plastics are produced annually, with substantial portions ending up in landfills or oceans, contributing to severe pollution issues [1]. The construction industry, which heavily relies on concrete and masonry materials, presents a viable sector for recycling plastic waste, transforming it into valuable construction components.

Concrete is the second most used material in the world [2]. Cement companies are responsible for 8% of the world’s carbon dioxide emissions, posing a significant environmental threat [3]. Utilizing waste materials in concrete is gaining popularity as it can reduce product costs and provide environmental benefits [4,5,6,7,8,9,10].

Concrete masonry blocks can replace traditional stones and bricks. They offer benefits such as lower cost, lighter weight, easier transport, faster construction, and uniform quality. They can be used in various locations, including interior walls, ceilings, and exterior walls, consuming less space.

With increasing populations and construction demands, the need for eco-friendly materials has led researchers to explore alternatives like post-consumer plastics, specifically PET. Studies demonstrate that incorporating recycled plastic in concrete can reduce environmental impact while improving certain mechanical properties. Research has shown that incorporating plastic waste into concrete can enhance its properties while mitigating environmental impacts [11,12,13,14,15,16,17,18,19,20,21,22]. For instance, Al-Tulaian et al. [23] showed that recycled plastic fibers enhance the durability of Portland cement mortar, contributing to improved mechanical properties and longevity. Marthong and Sarma [24] found that the geometry of PET fibers significantly improves both strength and ductility in concrete, illustrating the role that fiber shape can play in material performance. Islam and Shahjalal [25] investigated the partial replacement of natural stone aggregates with polypropylene (PP), observing that while the slump value increased with higher PP content, both compressive strength and modulus of rupture decreased. This highlights the trade-off between workability and strength when using PP in concrete. Similarly, studies by Belmokaddem et al. [26] and Kurup and Kumar [27] highlighted the potential of plastic waste to improve the thermal, acoustic, and hardened properties of concrete. Kan and Demirboga et al. [28] focused on using modified waste expanded polystyrene aggregates (MEPS), obtained by heat-treating EPS foam at 130 °C for 15 min, to replace natural aggregate in lightweight concrete. As the MEPS volume increased from 0% to 100%, the concrete became lighter, but its compressive strength decreased, reaching a minimum of 12.58 MPa with 100% replacement. Despite the lower strength, the concrete still met the requirements for semi-structural lightweight members. Moreover, Ozbakkaloglu et al. [29] studied the effect of recycled polypropylene as a partial replacement of fine aggregates. Results revealed that the compressive strength, tensile strength, and modulus of elasticity all decrease with the increase in PP percentage.

Research on PET specifically has shown that its incorporation into concrete improves load-bearing capacity, ductility, and energy dissipation. Marthong et al. [30] investigated the effect of incorporating PET plastic in the exterior reinforced concrete. Results show that PET enhances the load-resisting capacity, stiffness, displacement of ductility, and energy dissipation potential. Similarly, Foti [31] found that the inclusion of PET in concrete increases the ductility of concrete and enhances the adherence to mixing. The study found that PET delays the cracks in the member, and it improves the resistance, mechanical strength, and ductility of concrete. Similarly, Irwan et al. [32] studied the effect of partially replacing fine aggregate with PET plastic with a ratio of 25%, 50%, and 75%. The results showed that the inclusion of PET decreases the initial load cracks respectively to 27%, 38%, and 46% in comparison to the concrete without PET. This has an impact on the shear resistance value of the member. Kim et al. [33] investigated the behavior of structural members including PET and PP plastic. Results revealed that the behavior of beams including PET and PP is similar. The compressive strength and the elastic modulus both decrease with the increase in plastic percentage. However, beams containing PET fiber showed delayed cracking, greater ductility, and higher ultimate strength compared to beams without PET fiber, indicating that PET fiber improved the performance of concrete in structural applications.

The impact of plastic waste on concrete has been explored primarily as a replacement for sand or cement [34,35,36,37,38,39]. Few studies have investigated the impact of using polyethylene terephthalate (PET) as a partial replacement for coarse aggregates specifically in unreinforced concrete such as masonry blocks. This study explores the feasibility of using shredded PET plastic as a partial replacement for coarse aggregates to be used in masonry blocks. It aims to evaluate how this substitution impacts the structural integrity and durability of the blocks as well as the thermal conductivity of the new material. The use of shredded PET not only addresses the issue of plastic waste but also contributes to the sustainability of the construction industry by enhancing the environmental and functional performance of masonry blocks contributing to United Nations Sustainable Development Goal 12 (Responsible Consumption and Production) through the promotion of recycling and sustainable material usage.

## 2. Materials and Methods

### 2.1. Materials

**Figure 1:**
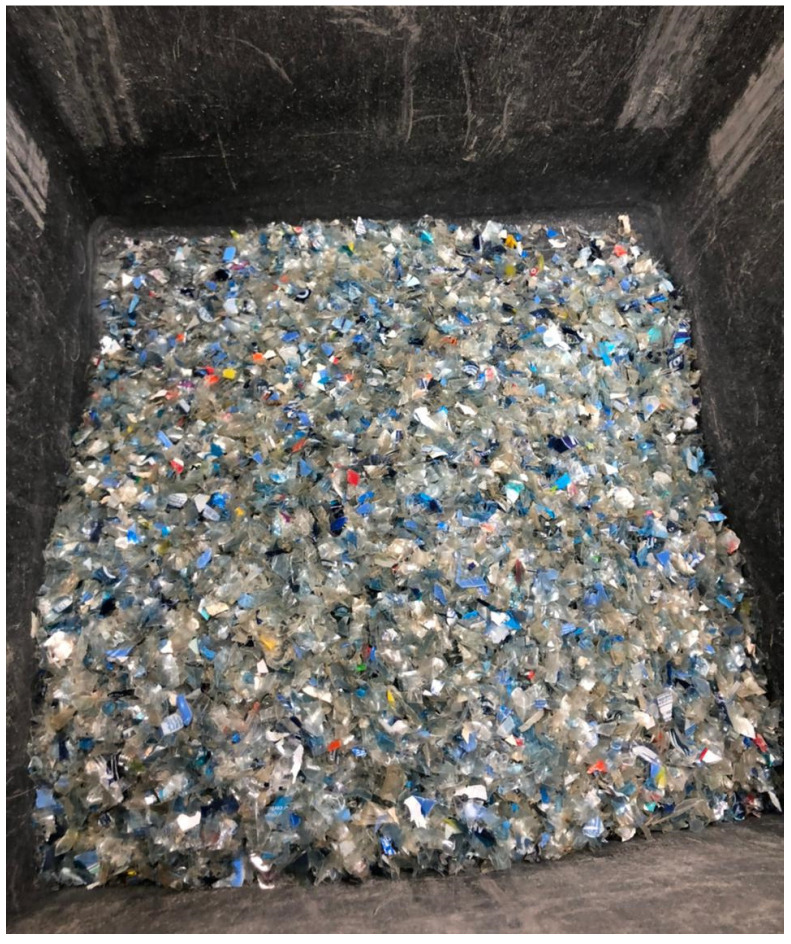
Shredded PET flakes.

The study aims to evaluate the feasibility of using shredded plastic as a replacement for traditional aggregates in unreinforced concrete, with a particular focus on how this substitution impacts the structural integrity and durability of the blocks. The PET used in this study was sourced from local recycling centers, specifically post-consumer PET bottles, which are representative of typical waste generated by households and businesses. The bottles were then thoroughly cleaned to remove any residues that could negatively impact the concrete’s performance. The cleaned plastic was subsequently shredded into 4–5 mm particles and an average thickness of 0.5 mm using a flake type Crusher GP500 machine, ensuring uniformity in size for consistency in the mixture. Figure 1 shows the shredded PET flakes.

The materials used in the blocks included Ordinary Portland Cement (OPC), which was selected to ensure high quality and consistency. Potable water was used to avoid impurities that could affect the setting and strength of the concrete. The physical properties of the coarse aggregates were determined using sieve analysis and the Los Angeles abrasion test. Sieve analysis provided a detailed distribution of particle sizes, while the abrasion test evaluated the aggregates’ durability and resistance to degradation. The study used C20 grade concrete with a Water/Cement (W/C) ratio of 0.5, and the required components for 1 m^3^ of the concrete mix are detailed in Table 1. The mixing process begins by first combining water and cement to form a cement paste. Once the paste is achieved, natural aggregates are introduced and mixed at a low torque for 2 min to ensure even distribution. PET aggregates are added to the mix and blended at the same low torque for an additional 2 min, carefully incorporating the PET particles into the matrix. The torque is then increased to a moderate level for 3 min to guarantee full integration of all materials. Finally, the mix is subjected to a final round of low torque blending for 2 min, resulting in a smooth and uniform mixture ready for placement.

**Figure 2:**
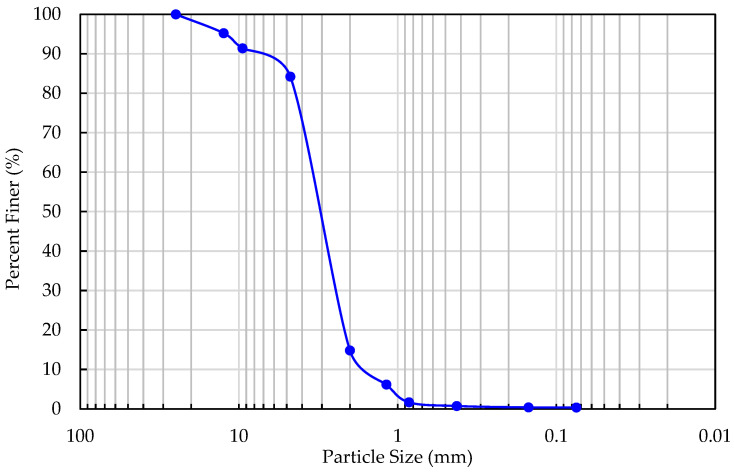
Particle size distribution curve of sand.

The fineness modulus (FM) is crucial for determining the ratio of fine to coarse aggregates when formulating concrete mixes. FM is defined as the percentage obtained from the sum of the cumulative percentages retained on the 150, 300, and 600 μm, and 1.18, 2.36, and 4.75 mm sieves and up to the largest size used [40]. ASTM C33/C33M-18 specifies that the fineness modulus of sand should not fall below 2.3 or exceed 3.1, to ensure that the sand is neither too fine nor too coarse [41]. The particle size distribution curve of sand used in this study is shown in Figure 2. The calculated FM is equal to 2.82 showing that the used sand has characteristics consistent with high-quality standards for aggregate materials.

Regarding the Los Angeles test, at the start of the experiment, 5 kg of gravel was used. The combined weight of the container and the gravel retained on the sieve 1.7 mm was recorded as 4.513 kg, with the container itself weighing 0.481 kg and the retained gravel weighing 4.032 kg. The weight of the gravel that passed through the sieve was calculated to be 0.968 kg. The Los Angeles (LA) abrasion value was calculated to be 20%, placing the gravel within the “Good to medium” category according to the Los Angeles test classification. This classification confirms that the gravel is suitable for use in the mixture [41].

**Figure 3:**
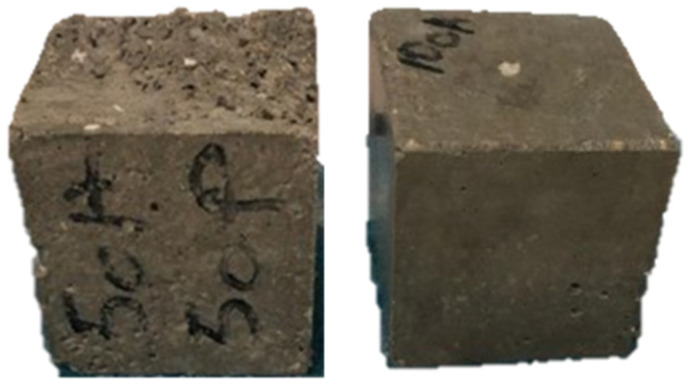
Samples of concrete blocks.

Fifty standard blocks, each with dimensions of 100 × 100 × 100 mm, were produced, along with five beam specimens, each measuring 100 × 100 × 500 mm. Different samples were developed by varying the amount of plastic in the aggregate mixture, replacing coarse aggregates with mass with ratios of 0%, 5%, 25%, 35%, and 50%. This variety allowed the study to analyze the impact of different levels of plastic content on the concrete blocks’ properties. Figure 3 illustrates samples from the cast concrete blocks.

### 2.2. Experimental Tests

A series of tests were performed on the samples to assess their physical and performance characteristics under controlled laboratory conditions. Temperature was maintained at 23 ± 5 °C and a relative humidity of 50 ± 10%. To ensure the reliability of the results, each test was conducted on five samples.

#### 2.2.1. Slump Test

The slump test is used to assess the concrete’s workability, indicating the ease of its placement and compaction. The ASTM C143/C143M-12 slump test measures the workability of fresh concrete by observing how it slumps when a cone-shaped mold is removed [42]. The test is effective for concrete with slumps between 1 and 6 inches (25 to 150 mm), indicating moderate workability. Concrete with a slump of less than 1/2 inch (15 mm) may be too stiff and lack adequate plasticity for the test to be meaningful, while a slump over 9 inches (230 mm) suggests excessive fluidity, which can lead to segregation and poor cohesion, rendering the test results unreliable.

#### 2.2.2. Air Content Test

The air content test is used to measure the volume of air voids in freshly mixed concrete to ensure the durability and strength of the concrete. The percentage of air contained in a sample of freshly mixed concrete is measured using Press-Aire™ Meter, model LA-0316. This model has a capacity of 7 L and includes an air pump, precision pressure gauge, and valves. The air content range is 0–100%, with gauge graduations of 0.1% up to 6% and 0.2% from 6–10%. The test is based on the principle that the total volume of a given sample of concrete is made up of the volume of the solid particles, the volume of the water, and the volume of the air.

#### 2.2.3. Compressive Strength Test

**Figure 4:**
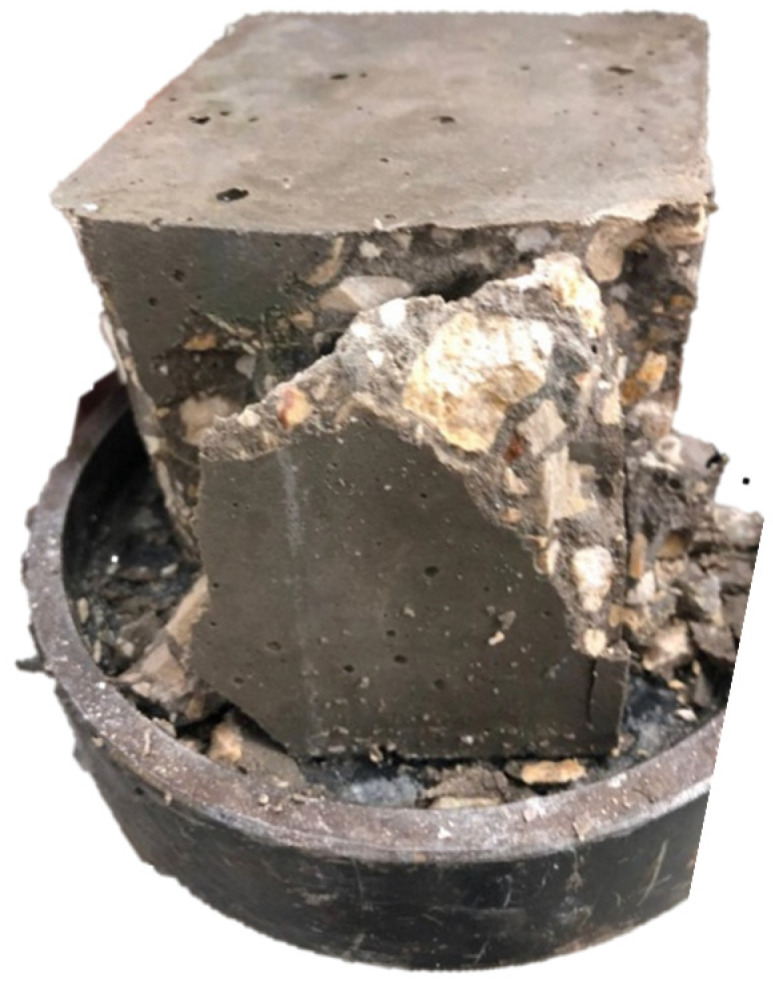
Failure of the concrete block under compression test.

The compressive strength test is necessary to measure the ability of a material to withstand loads. It is a key indicator of material performance in structural applications, influencing the durability and stability of buildings and infrastructure. The compressive strength values of the concrete samples are determined through standardized testing [43], using the PILOT PRO 50-C92C22 compression testing machine with a 600 kN capacity, where a sample was subjected to increasing compressive force until it failed as shown in Figure 4. The resulting maximum load divided by the sample’s cross-sectional area provides compressive strength.

#### 2.2.4. Flexural Strength Test

**Figure 5:**
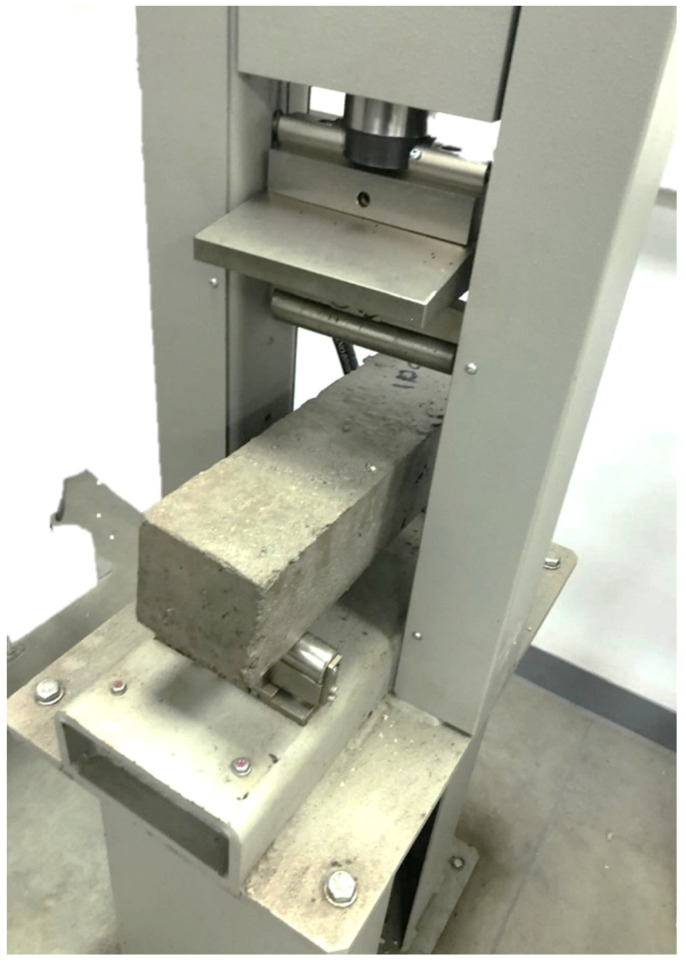
Flexural strength test setup for a beam specimen.

The flexural strength test is used to evaluate the concrete’s ability to resist bending or flexural stresses. This indicates its load-bearing capacity and durability under bending forces. The flexural strength values of the concrete samples were determined using a flexural frame PILOT PRO 50-C0910/FR with a capacity of 100 kN through standardized testing ASTM C293/C293M-16 [44], where a beam specimen was subjected to a gradually increasing load until it failed as shown in Figure 5.

#### 2.2.5. Thermal Conductivity Test

**Figure 6:**
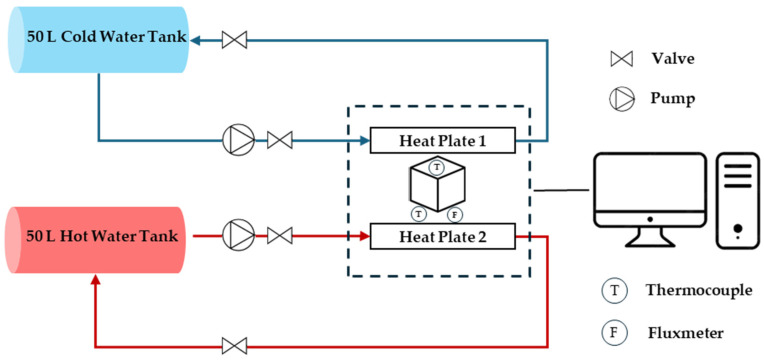
Schematic representation of the thermal experimental setup.

The thermal conductivity test provides data on the blocks’ insulation properties, essential for evaluating their performance in building applications. The thermal characterization is based on the flux metric method (NF EN 12939) [45]. It is a custom-made water circulation system, with two circuits pumping from a 50 °C water vessel and another vessel at room temperature. The hot and cold water are in contact with opposite sides of the test block using two heat plates equipped with a thermocouple and flux meter to measure the energy flux and the temperatures at each side. The fluxmeter and the two thermocouples are connected to a data acquisition of an electronic system to record the evolution of the heat flux φ (W/m^2^) and the temperatures T_1_ (°C) and T_2_ (°C). All the experimental components are placed in an insulated chamber and the tested samples are enclosed laterally with mineral wool insulation material in order to decrease the lateral heat losses and ensure unidirectional heat transfer. The fluxmeter used in this experiment is the HPF01 with a sensitivity of 62.51 µV.W^−1^.m^−2^. The sensor consists of a plate of 80 mm in diameter, a thickness of 5 mm, and a weight of 200 g excluding the cable. The temperature range for this sensor is between −30 °C and +70 °C, with a measurement range of ±2000 W/m^2^. Figure 6 shows an illustration of this setup.

The thermal resistance R (m^2^.°K/W) of the sample is calculated using the equation below. This has to be calculated once the temperatures and heat flow stabilize and reach convergence.

(1) $$R=\frac{T_{1}-T_{2}}{\varphi }$$

For a sample with a thickness e (m), the thermal conductivity λ (W/m.°K) can be deduced using the following equation:

(2) $$\lambda =\frac{e}{R}$$

## 3. Results

The following section presents the experimental results of various tests conducted to evaluate the performance characteristics of concrete blocks incorporating PET plastic waste as a partial replacement for coarse aggregate. The properties assessed include workability, air content, density, compressive strength, flexural strength, and thermal conductivity. These properties are important in determining the suitability of the modified concrete for specific construction applications, particularly in masonry where the balance of strength and workability is essential.

### 3.1. Slump Test Results

**Figure 7:**
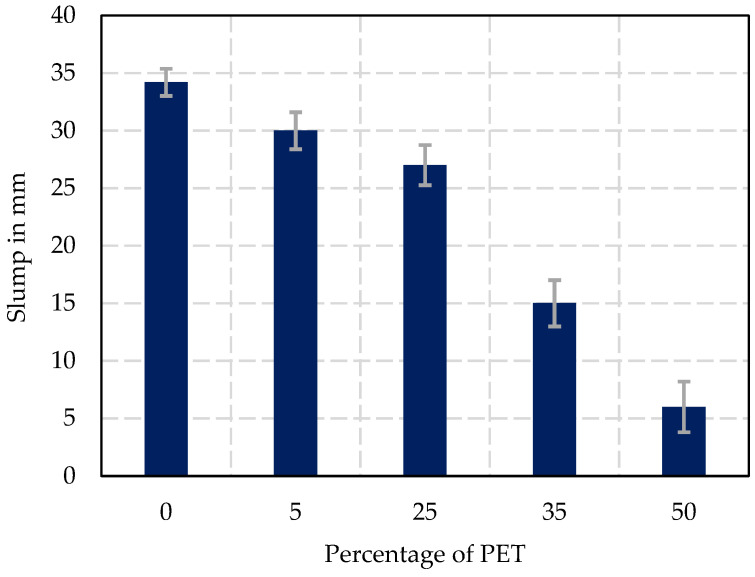
Slump test results according to the percentage of PET replacement.

The results of the slump test, shown in Figure 7, indicate a progressive decrease in workability as the percentage of PET replacement increases. The control mix (0% PET) showed the highest slump of 34 (±1.2) mm, reflecting optimal workability for a standard concrete mix. Introducing PET at 5% replacement slightly reduced the slump to 30 (±1.6) mm, indicating minimal impact on workability at this level. As the PET content increased to 25%, the slump further reduced to 27 (±1.7) mm, indicating that while the workability remains acceptable for construction purposes, the plasticity of the mix is gradually decreasing. At 35% PET replacement, the slump dropped significantly to 15 (±2) mm, indicating a much stiffer mix with poor workability. Finally, at 50% PET replacement, the slump was measured at only 6 (±2.2) mm, reflecting very stiff concrete with minimal workability. These results suggest that small amounts of PET (up to 25%) can be integrated without severely compromising workability, while higher percentages (35% and above) lead to significant reductions in a slump, indicating the concrete mix becomes less fluid and harder to handle.

### 3.2. Air Content Test Results

**Figure 8:**
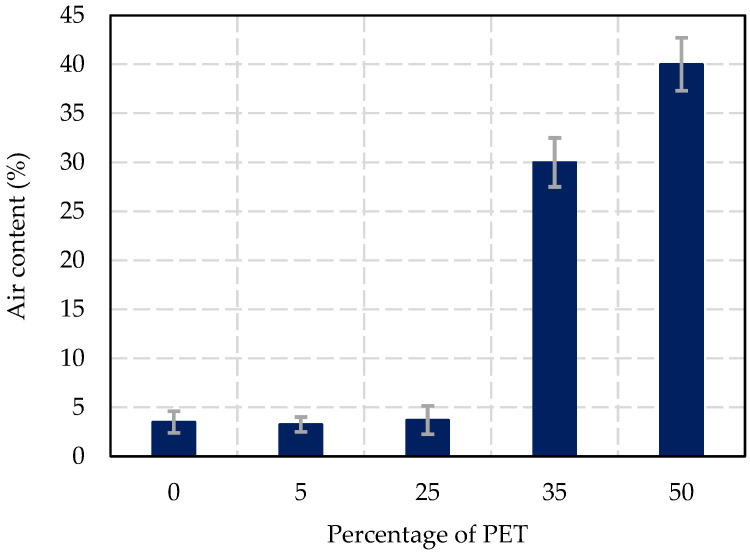
Air content results according to the percentage of PET replacement.

The air content values for fresh concrete with different percentages of PET replacement are shown in Figure 8. The results demonstrate a correlation between PET replacement and the increased porosity in the concrete mix. For the control sample (0% PET), the air content was measured at 3.5 (±1.1)%. At 5% and 25% PET replacement, the air content slightly fluctuated, with values of 3.3 (±1)% and 3.7 (±1.4)%, respectively. These minor variations indicate that low levels of PET substitution have a negligible effect on the air content of the concrete, allowing the material to retain structural properties suitable for masonry applications. However, when the PET content was increased to 35%, the air content reached 30 (±2.5)%, indicating a significant increase in voids within the concrete matrix. At 50% PET replacement, the air content further increased to 40 (±2.7)%, suggesting that higher levels of PET lead to a highly porous concrete mix. This increase in porosity is expected to negatively affect the concrete’s mechanical properties, such as strength and durability, but could enhance properties like insulation and sound absorption, making it suitable for non-structural applications.

### 3.3. Density Results

**Figure 9:**
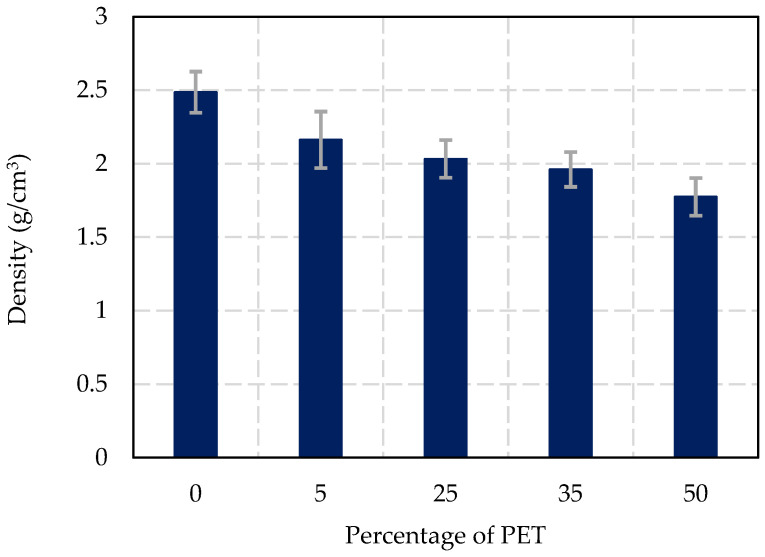
Variation of density in function of to the percentage of PET replacement.

As shown in Figure 9, the density of concrete decreases consistently with increasing PET replacement. The control sample (0% PET) presented the highest density at 2.49 (±0.1) g/cm^3^, reflecting the standard density of concrete with natural coarse aggregates. At 5% PET replacement, the density slightly decreased to 2.16 (±0.2) g/cm^3^, indicating a minimal reduction in overall mass. At 25% PET replacement, the density further decreased to 2.03 (±0.1) g/cm^3^, showing a moderate reduction in the overall weight of the concrete. This trend continued as PET replacement increased to 35%, with a corresponding density of 1.96 (±0.1) g/cm^3^. Finally, at 50% PET replacement, the density was reduced to 1.77 (±0.1) g/cm^3^, highlighting a significant decrease in the mass of the concrete.

This reduction in density is a result of replacing heavier natural aggregates with lighter PET plastic waste. While this leads to lighter concrete, which may be advantageous in applications where weight reduction is critical, the lower density also indicates a loss in structural integrity, making the material more suitable for non-load-bearing applications.

### 3.4. Compressive Strength Test Results

**Figure 10:**
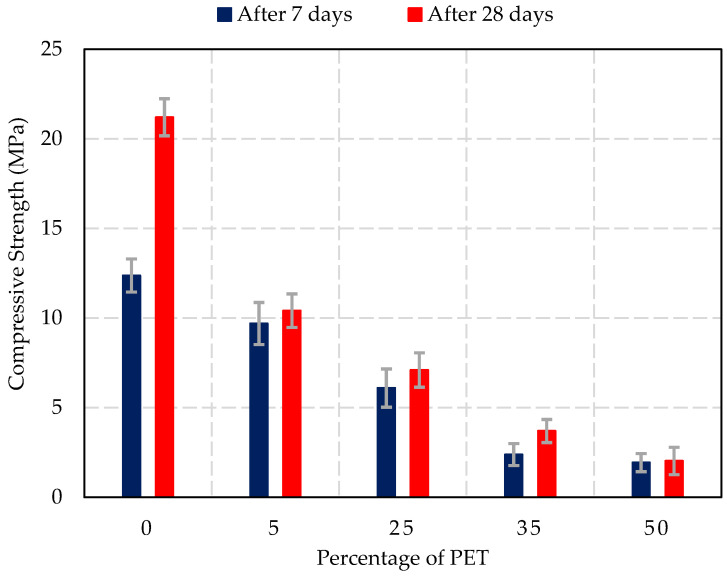
Effect of PET percentage on compressive strength of concrete blocks.

Figure 10 shows the compressive strength values of the concrete blocks at different percentages of PET replacement after 7 days and 28 days of curing.

The compressive strength results show a clear decrease in strength as the percentage of PET replacement increases. After 7 days of curing, the control sample (0% PET) achieved a compressive strength of 12.4 (±0.9) MPa, which further increased to 21.2 (±1) MPa after 28 days of curing, reflecting the expected strength development for standard concrete. At 5% PET replacement, the compressive strength after 7 days decreased to 9.7 (±1.1) MPa, with a corresponding value of 10.4 (±0.9) MPa after 28 days. This indicates a moderate reduction in strength. At 25% PET replacement, the compressive strength after 7 days was 6.1 (±1) MPa, and 7.1 (±1) MPa after 28 days, suggesting that the strength has been significantly impacted by the presence of PET.

For higher levels of PET replacement, the compressive strength dropped more drastically. At 35% PET, the strength after 7 days was 2.4 (±0.6) MPa and after 28 days was 3.7 (±0.6) MPa, barely meeting the ASTM C129-23 standard for non-load-bearing masonry units (3.45 MPa) [46]. Finally, at 50% PET replacement, the compressive strength fell to 1.9 (±0.5) MPa after 7 days and 2 (±0.8) MPa after 28 days, well below the standard requirements for most construction applications. These results suggest that PET replacement above 25% significantly compromises the compressive strength of concrete, limiting its use to non-structural or low-strength applications.

### 3.5. Flexural Strength Test Results

**Figure 11:**
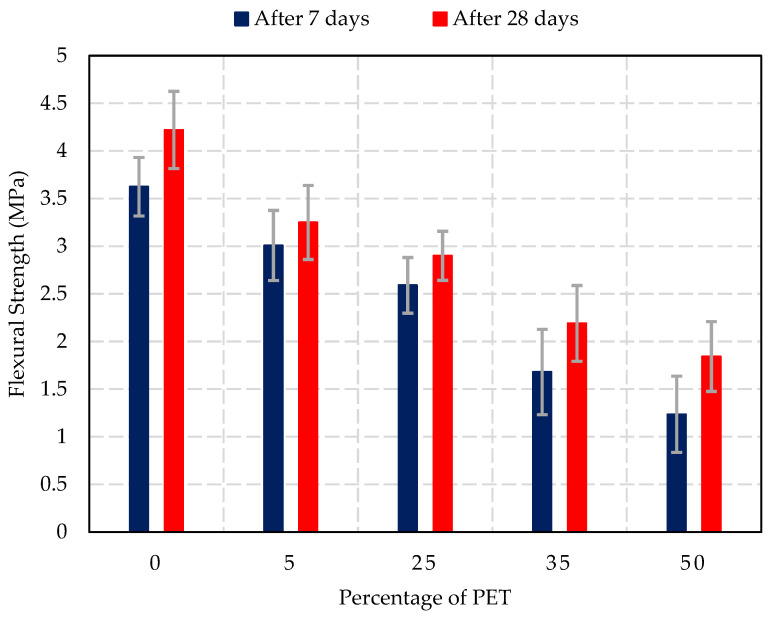
Effect of PET percentage on flexural strength of concrete blocks.

The results of the flexural strength test for concrete blocks after 7 and 28 days of curing with varying percentages of PET replacement are shown in Figure 11. The experimental tests show a significant decrease in flexural strength as the percentage of PET increases. At 0% PET, the flexural strength is highest, with 3.6 (±0.3) MPa after 7 days and 4.2 (±0.4) MPa after 28 days, which aligns with the typical range for normal-weight concrete [47]. As PET replacement increased to 5%, the flexural strength after 7 days was measured at 3 (±0.4) MPa, and after 28 days at 3.3 (±0.4) MPa, showing a slight reduction in tensile strength. At 25% PET replacement, the flexural strength further decreased to 2.6 (±0.3) MPa after 7 days and 2.9 (±0.3) MPa after 28 days. This indicates a more significant loss in resistance to bending forces. For higher PET replacement levels, the flexural strength values dropped significantly. At 35% PET, the strength after 7 days was 1.7 (±0.4) MPa, and after 28 days, it was 2.2 (±0.4) MPa. At 50% PET replacement, the flexural strength was lowest at 1.2 (±0.4) MPa after 7 days and 1.8 (±0.4) MPa after 28 days, indicating that the material’s ability to withstand bending forces is severely compromised at high PET levels.

### 3.6. Thermal Conductivity Test Results

Based on the results of the slump test, air content test, density measurements, and compressive and flexural strength tests summarized in Table 2, the sample with 25% PET replacement is identified as having a balanced performance. This sample is characterized by moderate workability with a slump value of 27 mm, minimal impact on air content with a value of 3.7%, and relatively high compressive strength of 7.1 MPa after 28 days of curing. These properties suggest its suitability for masonry walls while retaining adequate structural integrity. Consequently, this sample was selected for the thermal conductivity test to further evaluate its potential for use in construction.

**Figure 12:**
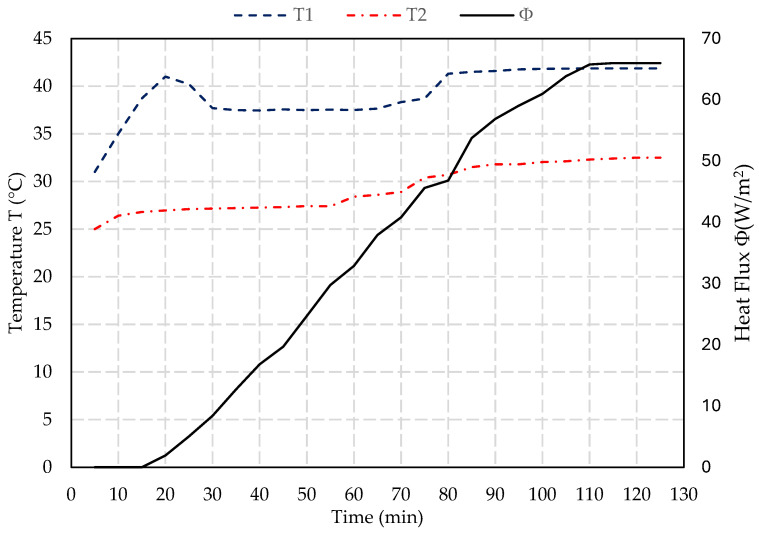
Variation of temperature and heat flux in the function of time for a concrete block with 0% PET.

**Figure 13:**
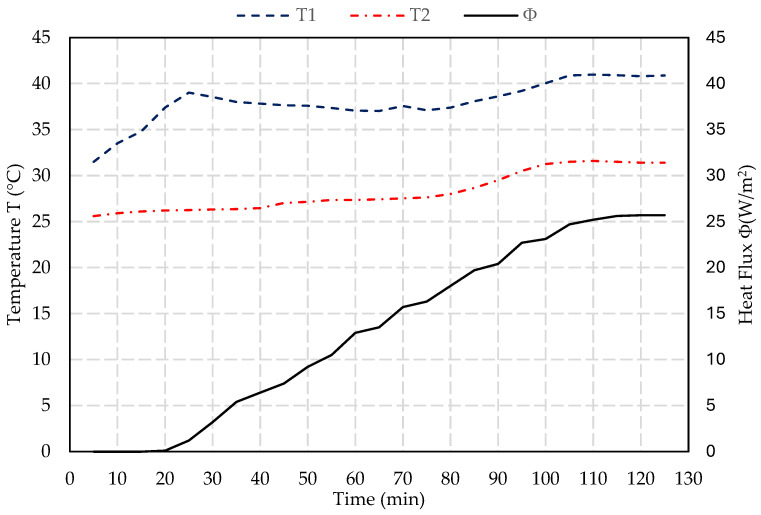
Variation of temperature and heat flux in the function of time for a concrete block with 25% PET.

As shown in Figure 12 and Figure 13, the steady state is reached after 2 h. Based on the measurements and Equations (1) and (2), the thermal conductivity for the sample with 0% PET is determined to be 0.7 W/m.°K as detailed in Table 3. This value is within the typical range for normal-weight concrete, which is between 0.6 and 3.3 W/m.°K [48,49]. For the sample with 25% PET, the thermal conductivity decreases to 0.27 W/m.°K, enhancing its insulation properties and making it significantly more effective at minimizing heat transfer than the sample with 0% PET. Incorporating the material with 25% PET into the building envelope, such as in walls, can significantly enhance the thermal efficiency of structures. It reduces energy consumption for heating and cooling, thereby lowering utility bills and improving indoor comfort.

## 4. Discussion

This section interprets the previously presented results. The implications of incorporating PET plastic waste as a partial replacement for coarse aggregate in concrete will be also discussed. Additionally, the findings are compared with relevant literature to provide context and validate the observed trends.

The significant reduction in slump with increasing PET content indicates a loss of workability due to the hydrophobic nature of PET, which does not interact well with water, leading to reduced lubricity in the mix. This is consistent with findings by Islam and Shahjalal [25] and Rahmani et al. [50], who reported similar reductions in workability with increased PET replacement. Higher PET levels could necessitate the use of superplasticizers or additional water to maintain desirable workability.

Increased air content at higher PET replacement levels, rising from 3.5% in the control mix to 40% at 50% PET replacement, suggests that PET particles create voids and trapped air, which could negatively affect density and strength in load-bearing applications. However, for non-structural elements where weight reduction is prioritized, this increase in porosity may be advantageous. The results show a clear and consistent reduction in density as PET replacement increases. This trend is primarily due to the significantly lower specific gravity of PET (1.38 g/cm^3^) compared to natural coarse aggregates (2.65 g/cm^3^). As more PET replaces natural aggregates, the overall mass of concrete decreases, resulting in a lighter material. This reduction in density may be beneficial for applications requiring lightweight materials, such as precast elements or insulation panels, as supported by Yang et al. [51]

The compressive strength results indicate a significant reduction in strength as the PET content increases, with values falling below the ASTM C90-23 standard for load-bearing units (13.8 MPa after 28 days) [52] at PET replacements above 25%. This reduction in strength can be attributed to the poor bonding between the non-porous PET particles and the cementitious matrix, which limits the concrete’s ability to bear loads. These findings are in agreement with previous research by Albano et al. [53] and Azhdarpour et al. [54], who observed a similar decrease in compressive strength when PET was used as a replacement for natural aggregates in concrete. It is expected that the weak bond between PET and the cement paste, along with the increased air voids introduced by PET particles, is the primary cause of this reduction in strength. Although the compressive strength declines significantly with increasing PET content, concrete with up to 25% PET replacement still shows sufficient strength for non-load-bearing applications, such as masonry units. Beyond this level, the reduction in strength becomes too significant for structural applications, limiting the use of PET-modified concrete to specific non-structural or lightweight applications.

The flexural strength results follow a similar trend to the compressive strength, with significant reductions observed as PET content increases. This decrease in tensile strength can be attributed to the brittle nature of PET particles and their poor interaction with the surrounding cement paste. PET’s inability to form strong interfacial bonds with the cement matrix leads to weaker concrete, which is more prone to cracking and failure under tensile stresses. Frigione [4] and Saikia et al. [55] observed similar reductions in flexural strength when incorporating plastic waste into concrete. The research suggested that plastic particles act as stress concentrators, which leads to the early initiation of cracks and reduced resistance to bending forces. Despite these challenges, PET-modified concrete with up to 25% replacement remains adequate flexural strength for non-structural applications, such as non-load-bearing walls.

The results of the thermal conductivity tests indicate that incorporating PET into the concrete mix significantly improves its insulation properties. The reduction in thermal conductivity from 0.7 W/m·K for the control mix to 0.27 W/m·K for the mix with 25% PET replacement highlights the potential of PET-modified concrete as an insulating material. These findings are in line with research conducted by Poonyakan et al. [56], who found that plastic-modified concrete showed higher thermal insulation properties compared to traditional concrete. The low thermal conductivity of PET particles contributes to this improvement, making PET-modified concrete suitable for applications where energy efficiency is prioritized. Such applications could include wall panels, roof insulation, or façade systems in energy-efficient buildings.

While the current study focuses on laboratory testing and achieves Technology Readiness Level 4 (TRL4), further research is essential to fully assess the practical applications of PET-modified masonry blocks. Future studies should include numerical simulations to predict the long-term behavior of these materials under various loading conditions and environmental factors. Moreover, in situ testing on full-scale buildings or components is necessary to validate the laboratory findings in practical settings. This could include testing masonry blocks in real construction environments to evaluate their structural capacity, thermal insulation performance, and durability under actual service conditions.

## 5. Conclusions

The sustainable integration of recycled materials, particularly shredded PET plastic, in unreinforced concrete is vital for reducing environmental impacts, conserving natural resources, and advancing eco-friendly building practices. This study has shown that incorporating up to 25% shredded PET plastic as a partial replacement for traditional coarse aggregates not only minimizes waste but also addresses the increasing demand for newly extracted materials, thereby lowering the carbon footprint of the construction industry.

The experimental results indicate that unreinforced concrete containing 25% PET maintains adequate structural integrity and meets the necessary performance criteria for non-load-bearing applications. Reasonable compressive strength, satisfactory density, and improved thermal insulation properties were demonstrated by this concrete compared to traditional mixes, suggesting significant potential for energy efficiency in building applications. Moreover, the use of this material in masonry blocks can further enhance sustainable construction efforts. However, it is crucial to note that exceeding the 25% PET replacement level leads to significant reductions in both compressive and flexural strengths, making the concrete unsuitable for structural applications. Therefore, it is recommended that unreinforced concrete with a maximum of 25% PET content be used for non-load-bearing walls and as a viable option for masonry blocks, as it effectively balances environmental benefits with essential performance criteria. While this study emphasizes the potential for recycled PET in sustainable construction, it also highlights the need for standardized processing techniques to ensure material consistency and performance. Future research should explore the economic viability, regulatory compliance, and long-term durability of these materials to facilitate their adoption in mainstream construction practices. Key areas for further investigation include economic analysis, life cycle cost assessments, performance testing, and an evaluation of environmental impacts, processing challenges, supply chain logistics, and market demand. Addressing these factors will be essential to determine the overall feasibility and scalability of using recycled PET in concrete, ultimately supporting its effective adoption and integration into sustainable construction practices.

## Figures and Tables

**Table 1:** Concrete mix proportion for 1 m^3^ of the concrete mix grade C20.

Material	Mass (kg)
Cement	383
Fine aggregate (Sand)	664
Coarse aggregate (Gravel)	1185
Water	191.5

**Table 2:** Summary of all results with percentages of variations with respect to the control sample (0% PET).

	PET%	0%	5%	25%	35%	50%
Tests						
Slump (mm)		34 (±1.2)	30 (±1.6)	27 (±1.7)	15 (±2)	6 (±2.2)
Variation (%)			−12%	−21%	−56%	−82%
Air content (%)		3.5 (±1.1)	3.3 (±1.1)	3.7 (±1.4)	30 (±2.5)	40 (±2.7)
Variation (%)			−7%	+6%	+757%	+1043%
Density (g/cm^3^)		2.49 (±0.1)	2.16 (±0.2)	2.03 (±0.1)	1.96 (±0.1)	1.77 (±0.1)
Variation (%)			−13%	−18%	−21%	−29%
Compressive strength after 7 days (MPa)		12.4 (±0.9)	9.7 (±1.1)	6.1 (±1)	2.4 (±0.6)	1.9 (±0.5)
Variation (%)			−22%	−51%	−81%	−84%
Compressive strength after 28 days (MPa)		21.2 (±1)	10.4 (±0.9)	7.1 (±1)	3.7 (±0.6)	2 (±0.8)
Variation (%)			−51%	−67%	−83%	−90%
Flexural strength after 7 days (MPa)		3.6 (±0.3)	3.0 (±0.4)	2.6 (±0.3)	1.7 (±0.4)	1.2 (±0.4)
Variation (%)			−17%	−29%	−54%	−66%
Flexural strength after 28 days (MPa)		4.2 (±0.4)	3.3 (±0.4)	2.9 (±0.3)	2.2 (±0.4)	1.8 (±0.4)
Variation (%)			−23%	−31%	−48%	−56%

**Table 3:** Results of thermal conductivity tests for 0% and 25% PET replacement.

Sample	T_1_ (°C)	T_2_ (°C)	φ (W/m^2^)	R (m^2^K/W)	λ (W/m.°K)
0% PET	41.88	32.50	66.0	0.14	0.70
	(±0.5)	(±0.7)	(±3.3)	(±0.007)	(±0.05)
25% PET	40.88	31.40	25.7	0.37	0.27
	(±0.7)	(±0.8)	(±4.2)	(±0.06)	(±0.05)

## Data Availability

The original contributions presented in the study are included in the article, further inquiries can be directed to the corresponding author.
